# Association Genetics in *Populus* Reveal the Allelic Interactions of *Pto-MIR167a* and Its Targets in Wood Formation

**DOI:** 10.3389/fpls.2018.00744

**Published:** 2018-06-12

**Authors:** Mingyang Quan, Liang Xiao, Wenjie Lu, Xin Liu, Fangyuan Song, Jingna Si, Qingzhang Du, Deqiang Zhang

**Affiliations:** ^1^Beijing Advanced Innovation Center for Tree Breeding by Molecular Design, College of Biological Sciences and Technology, Beijing Forestry University, Beijing, China; ^2^National Engineering Laboratory for Tree Breeding, College of Biological Sciences and Technology, Beijing Forestry University, Beijing, China; ^3^Key Laboratory of Genetics and Breeding in Forest Trees and Ornamental Plants, Ministry of Education, College of Biological Sciences and Technology, Beijing Forestry University, Beijing, China

**Keywords:** association genetics, *Pto-miR167a*, auxin response factor, long non-coding RNA, epistasis, wood formation, *Populus*

## Abstract

MicroRNAs (miRNAs) play crucial regulatory roles in plant growth and development by interacting with RNA molecules, including messenger RNAs (mRNAs) and long non-coding RNAs (lncRNAs); however, the genetic networks of miRNAs and their targets influencing the phenotypes of perennial trees remain to be investigated. Here, we integrated expression profiling and association analysis of underlying physiology and expression traits to dissect the allelic variations and genetic interactions of *Pto-MIR167a* and its targets, sponge lncRNA *ARFRL*, and *Pto-ARF8*, in 435 unrelated individuals of *Populus tomentosa*. Tissue-specific expression analysis in eight tissues, including stem, leaf, root, and shoot apex, revealed negative correlations between *Pto-MIR167a* and lncRNA *ARFRL* and *Pto-ARF8* (*r* = −0.60 and −0.61, respectively, *P* < 0.01), and a positive correlation between sponge lncRNA *ARFRL* and *Pto-ARF8* (*r* = 0.90, *P* < 0.01), indicating their potential regulatory roles in tree growth and wood formation. Single nucleotide polymorphism (SNP)-based association studies detected 53 significant associations (*P* < 0.01, *Q* < 0.1) representing 41 unique SNPs from the three genes and six traits, suggesting their potential roles in wood formation. Epistasis uncovered 88 pairwise interactions for 10 traits, which provided substantial evidence for genetic interactions among *Pto-MIR167a*, lncRNA *ARFRL*, and *Pto-ARF8*. Using gene expression-based association mapping, we also examined SNPs within the three genes that influence phenotypes by regulating the expression of *Pto-ARF8*. Interestingly, SNPs in the precursor region of *Pto-MIR167a* altered its secondary structure stability and transcription, thereby affecting the expression of its targets. In summary, we elucidated the genetic interactions between *Pto-MIR167a* and its targets, sponge lncRNA *ARFRL*, and *Pto-ARF8*, in tree growth and wood formation, and provide a feasible method for further investigation of multi-factor genetic networks influencing phenotypic variation in the population genetics of trees.

## Introduction

MicroRNAs (miRNAs) are a class of non-coding RNAs (ncRNAs) that are derived from hairpin precursors and loaded into the RNA-induced silencing complex to post-transcriptionally regulate gene expression via cleavage or inhibitory mechanisms (Ramachandran and Chen, 2008; Voinnet, 2009). Evidence suggests that miRNAs have emerged as master regulators of plant development, physiological responses, and resistance to biotic and abiotic stresses (He and Hannon, 2004; Sunkar et al., 2012). In plants, increasing numbers of miRNAs implicated in plant growth and development have been identified in multiple species (Kozomara and Griffiths-Jones, 2014), including *Arabidopsis* (Ramachandran and Chen, 2008) and *Populus* (Chen et al., 2016). Of these, miR167 was found to be involved in significant biological processes by interacting with its target genes, *auxin response factors* (*ARFs*). For example, nitrogen treatment increases the expression of *ARF8* by reducing miR167 levels in the pericycle and root cap, initiating lateral root formation in *Arabidopsis* (Gifford et al., 2008). Additionally, miR167 is essential for both ovule and anther development by regulating the expression patterns of *ARF6* and *ARF8* in *Arabidopsis* (Wu et al., 2006). In perennial trees, miR167 also represents a class of miRNAs that respond to stresses such as temperature, mechanical stress, and ultraviolet-B radiation (Lu et al., 2008; Jia et al., 2009). However, the detailed regulatory mechanisms and how the interactions between miR167 and *ARFs* contribute to tree growth and development, remain largely unknown.

Recently, an additional layer of regulation affecting miRNA accumulation involving miRNA sponge was proposed (Ebert and Sharp, 2010). Sponge RNAs contain miRNA binding sites, and regulate the expression of corresponding genes by competing for interactions with miRNA. In plants, long non-coding RNAs (lncRNAs) serve as an important type of sponge RNA, modulating the expression of miRNA targets (Franco-Zorrilla et al., 2007). LncRNAs are a class of crucial non-coding transcripts that have been implicated in many aspects of biological processes in plants and animals (Ponting et al., 2009; Quan et al., 2015), such as photoperiod-sensitive male sterility in rice (Ding et al., 2012) and flowering time regulation in *Arabidopsis* (Swiezewski et al., 2009; Heo and Sung, 2011). The endogenous lncRNA *Induce by Phosphate Starvation 1* (*IPS1*) in *Arabidopsis* contains a complementary motif to miR399, and *IPS1* alters the stability of *PHOSPHATE2* (*PHO2*) by sequestering miR399, the phosphate starvation induced miRNA. In *Populus*, few lncRNAs have been identified as miRNA sponges (Shuai et al., 2014; Tian et al., 2016b); nevertheless, the role of miRNA-lncRNA-mRNA crosstalk in phenotypic variation requires further investigation.

Perennial trees provide renewable materials for industry, such as timber resources, but they must adapt to complicated eco-environments (Neale and Kremer, 2011). Thus, investigating the genetic basis of tree growth and wood formation is critical for genetic breeding to improve the economic and ecological properties of trees. However, perennial woody plants possess long life cycles and lack characterized mutants, which hinders the use of transgenic approaches to examine the specific functions of miRNA targets. Due to the large population size and abundant genetic variants in the genome, single nucleotide polymorphism (SNP)-based association mapping has been applied as a feasible strategy for deciphering the allelic variations of traits in these plants (Neale and Savolainen, 2004). For example, Thumma et al. (2005) identified the alleles of the *cinnamoyl CoA reductase* (*CCR*) gene, a key gene in the lignin biosynthesis pathway that is significantly associated with the microfibril angel (MFA) of wood in *Eucalyptus nitens*. SNPs in miRNA genes have also been identified in humans using association studies, and SNPs in the precursor miRNA (pre-miRNA) region were found to affect miRNA secondary structure and expression (Bensen et al., 2013). Recently, the genetic interactions of miRNA-mRNA and lncRNA-mRNA underlying additive, dominance, and epistatic effects in tree growth and wood properties have been discussed (Chen et al., 2016; Tian et al., 2016b). Nevertheless, few studies have concentrated on the genetic interactions of miRNA-lncRNA-mRNA networks in tree growth and wood properties. Beyond that, association mapping of underlying expression traits could be used to identify genetic variants and expression phenotypes that ultimately contribute to phenotype diversification (Li et al., 2013), providing an alternative method for explaining the genetic effects of significant SNPs on traits at the transcriptional level. Thus, association studies of underlying expression traits aid in clarifying the comprehensive regulatory networks of miRNA-lncRNA-mRNA in tree growth and wood formation.

Here, we first identified a potential sponge lncRNA *auxin response factors-related lncRNA* (*ARFRL*) for *Pto-MIR167a-d*. Sequence alignment detected nucleotide variants in the pre-miRNA region of *Pto-MIR167a*, thus we cloned *Pto-MIR167a* for further analysis. Degradome sequencing and psRNATarget prediction identified *Pto-ARF8* as a target gene of *Pto-MIR167a* and lncRNA *ARFRL*. The regulatory roles of Pto-miR167a and its targets, lncRNA *ARFRL* and *Pto-ARF8*, were also supported by 5′ rapid amplification of cDNA ends (5′-RACE). Expression abundance analysis revealed a significant negative correlation between *Pto-MIR167a* and the targets lncRNA *ARFRL* and *Pto-ARF8*. Additionally, significant positive correlations were observed between lncRNA *ARFRL* and *Pto-ARF8*, indicating the potential role of lncRNA *ARFRL* as a Pto-miR167a sponge. Next, SNP-based association studies (additive, dominant, and epistatic) were conducted in an association population of 435 unrelated individuals of *P. tomentosa*, which deciphered the genetic interactions of SNPs within *Pto-MIR167a* and its targets, lncRNA *ARFRL*, and *Pto-ARF8*, underlying tree growth and wood properties. The association mapping of underlying expression traits led to an alternative hypothesis that SNPs in regulators (*Pto-MIR167a* and lncRNA *ARFRL*) and *Pto-ARF8* may affect phenotypic variation through regulating the expression of their target gene *Pto-ARF8*. Remarkably, we also found that SNPs in the pre-miRNA of *Pto-MIR167a* altered its secondary structure and expression. Our study provides a better understating of the genetic networks of *Pto-MIR167a* and its targets, lncRNA *ARFRL*, and *Pto-ARF8*, in tree growth and wood formation, and the association analysis of underlying physiology and expression traits proposed an alternative method for dissecting the genetic networks of ncRNAs and mRNAs in the population genetics of trees.

## Materials and methods

### Plant materials and phenotypes

The association population consisted of 435 unrelated individuals of *P. tomentosa* that represented almost the entire natural distribution of *P. tomentosa* (30–40°N, 105–125°E), i.e., southern, northwestern, and northeastern regions of China. Plants in this population were randomly selected from the *P. tomentosa* collection (1047 individuals) that was established in Guan Xian Country (Shandong province, China, 36°23′N, 115°47′E) in 1982, using a randomized complete block design approach with three clonal replications (Du et al., 2012). From this collection, 43 unrelated individuals of *P. tomentosa* were selected for SNP identification.

Ten growth and wood property traits were assessed across the 435 unrelated individuals with three replications per genotype. The growth traits were stem volume (V, m^3^), diameter at breast height (DBH, cm), and tree height (H, m). The wood property traits were MFA (°), fiber length (FL, mm), fiber width (FW, μm), lignin content (LC, %), holocellulose content (HC, %), α-cellulose content (CC, %), and hemicellulose content (HEC, %). Detailed measurement methods and phenotypic variations of the 10 traits have been previously reported (Du et al., 2014). Pearson's correlations for the 10 quantitative traits were calculated using SPSS Statistics v.19.0 (SPSS Inc., Chicago, IL, USA) (Table S1).

### Identification of *Pto-MIR167a* and its targets, lncRNA *ARFRL*, and *Pto-ARF8*

*Pto-MIR167a* was cloned based on the sequence of *Ptc-MIR167a* in *P. trichocarpa* (Kozomara and Griffiths-Jones, 2014) using gene-specific primers, including the pre-miRNA sequences and 1000-bp flanking sequences on each side of the pre-miRNA. The psRNATarget (http://plantgrn.noble.org/psRNATarget/) was used to predict the target genes of Pto-miR167a using 3000 mature xylem cDNA sequences from *P. tomentosa*. This cDNA library was constructed using the Superscript λ System (Life Technology, Carlsbad, CA, USA) according to manufacturer's instructions and consisted of 5.0 × 10^6^ plaque-forming units, with an insert size of 1.0–4.0 kb (Li et al., 2009). Then, degradome sequencing was used to identify the miRNA cleavage sites (Figure S1). Total RNA samples from six tissues, i.e., cambium, phloem, mature xylem, developing xylem, leaf, and shoot apex, were pooled together in equal amounts after RNA purification and integrity confirmation, and then used for degradome library construction and sequencing with the Illumina HiSeq2000, according to methods described previously (Shamimuzzaman and Vodkin, 2012). The CleaveLand pipeline (Addo-Quaye et al., 2009) was used to analyze the miRNA cleavage sites based on *P. trichocarpa* genome transcripts v.3.0 (Tuskan et al., 2006). Degradome sequencing identified a total of 596 miRNA-mRNA pairs, which are listed in Table S2 (Xie et al., 2017) (SRX1447192).

The target lncRNA of Pto-miR167a was also predicted by psRNATarget with the expectation ≤ 5.0. The lncRNA information was obtained from the transcriptome database of cambium, developing xylem, and mature xylem of *P. tomentosa* (SRP073689), as previously reported (Zhou et al., 2017). The prediction of lncRNA targets was based on sequence complementarity (*E*-value < 1e-5) and RNA duplex energy calculated by RNAplex with *E*-value < −60 (Tafer and Hofacker, 2008).

### 5′-race

RNA Ligase-Mediated 5′-RACE (RLM-RACE) was performed using SMARTer RACE Kit (TaKaRa, Dalian, China) according to the manufacturer's instructions with some modifications. PCR was performed using 5′-RACE CDS Primer A [5′-(T)_25_ V N-3′; N = A, C, G, or T; V = A, G, or C] and gene-specific primers (Table S3), using cDNA as the template. The products of RACE were gel-purified, cloned, and sequenced.

### Tissue-specific expression analysis

Eight fresh tissues, including phloem, cambium, developing xylem, mature xylem, root, shoot apex, old leaf, and young leaf, were harvested from 1-year-old *P. tomentosa* clone “LM50.” Total RNA was extracted from each tissue using a Plant Qiagen RNeasy kit (Qiagen China, Shanghai, China) according to the instructions. Additional on-column DNase digestions were conducted using an RNase-Free DNase Set (Qiagen) during the RNA purification. The cDNAs of the eight tissues were reverse transcribed using a Reverse Transcription System (Promega Corporation, Madison, WI, USA) following the manufacturer's instructions, and were then used to test the tissue-specific expression profiles. Reverse transcription quantitative PCR (RT-qPCR) was conducted with a 7500 Fast Real-Time PCR System, using SYBR Premix Ex Taq (TaKaRa). All reactions were conducted with triplicate technical and triplicate biological repetitions, using gene-specific primers designed with Primer Express 5.0 software (Applied Biosystems, Beijing, China) with *Actin* (EF145577) as the internal control (Table S4). The PCR program conditions were 94°C for 5 min, 40 cycles of 94°C for 30 s, 58°C for 30 s, and 72°C for 30 s, and a final melting curve from 70 to 95°C, which was used to confirm the specificity of the amplification. Opincon Monitor Analysis software 3.1 was used to analyze the data.

### SNP discovery and genotyping

Total genomic DNA was isolated from the fresh leaves of 435 unrelated individuals, using the DNeasy Plant Mini Kit (Qiagen China). To identify SNPs, the full-length genomic DNA of candidate genes was sequenced using gene-specific primers, which were designed based on the cDNA of candidate genes. The PCR amplification procedure has been described previously (Zhang et al., 2011). The BigDye Terminator Cycle Sequencing kit (version 3.1, Applied Biosystems) and the 4300 DNA Analyzer (Li-Cor Biosciences, Lincoln, NE, USA) were applied for sequencing. All 129 sequences of the three candidate genes were deposited in NCBI (https://www.ncbi.nlm.nih.gov/) under the accession numbers MG873890–MG873932 for *Pto-MIR167a*, MG873933–MG873975 for *Pto-ARF8*, and MG873976–MG874018 for *ARFRL*. Sequence alignment and SNP identification were conducted by MEGA 5.0 software (Tamura et al., 2011). Using the Beckman Coulter (Franklin Lakes, NJ, USA) sequencing system, the common SNPs (minor allele frequency [MAF] ≥ 5%) were genotyped across all 435 individuals in the association population (Table S5).

### Data analysis

#### Nucleotide diversity and linkage disequilibrium (LD) analysis

The nucleotide diversity was assessed based on π (Nei, 1987) and θ_w_ (Watterson, 1975), which were calculated using DnaSP 5.10 software (Librado and Rozas, 2009). The squared correlation of allele frequencies (*r*^2^) between each pair of common SNPs (MAF > 0.05) within the candidate genes was calculated by TASSEL 5.0 with 10^5^ permutations (Bradbury et al., 2007). The decay of LD with physical distance (bp) between each common SNP pair was estimated using non-linear regression (Remington et al., 2001), and singletons were excluded from the LD analysis.

#### Single SNP-based association analysis

SNP-trait associations were conducted using a mixed linear model (MLM) in TASSEL 5.0 (Bradbury et al., 2007), considering the pairwise kinship coefficients (K), and population structure (Q). The Q matrix was assessed using STRUCTURE v.2.3.4 (Patterson et al., 2006) based on significant subpopulations (*k* = 3), using the statistical model described by Evanno et al. (2005). The K matrix was evaluated with SPAGeDi 1.3 (Hardy and Vekemans, 2002) on the basis of 20 species-specific SSR markers (Du et al., 2012). The MLM equation was: y = μ + Qv + Zu + e, where y denoted the vector of phenotypic observations, μ denoted the intercept vector, v denoted the vector for population effects, u denoted the vector of random polygenic background effects, e denoted random experimental error, Q matrices denoted the population structure, and Z denoted the matrices relating y to u. Finally, corrections of the *P*-value for all the associations were performed by false discovery rate (FDR) using the QVALUE package in R (Storey and Tibshirani, 2003).

#### Haplotype-based association analysis

The high-LD haplotypes (*r*^2^ ≥ 0.75, *P* ≤ 0.01) were evaluated for each gene, and their frequencies were determined using Haploview v.4.2 (Barrett et al., 2005). The significance of haplotype-based associations was based on 10^4^ permutation tests by haplotype trend regression (HTR) (Zaykin et al., 2002). Singletons and haplotypes with frequencies less than 5% were excluded from our analyses.

#### Multi-SNP based epistasis analysis

The multifactor dimensionality reduction (MDR) algorithm was employed to detect the pairwise epistatic effects among the SNPs, for which high-dimensionality genetic data were processed into a single dimension to detect the non-additive interactions in a small set (Hahn et al., 2003). The ReliefF algorithm in MDR 3.0.2 filtered all the unlinked SNPs (*r*^2^ < 0.1 or different genes), and output the most significant SNPs for each trait after attribute selection, attribute construction, classification, and permutation testing. Entropy-based measures were performed for each SNP-SNP pair were performed and evaluated by information gain (IG) (Moore et al., 2006).

#### Gene expression-based association analysis

Gene expression-based association analysis interpreted the genetic effects of candidate loci for genes at the transcriptional level, which we performed at the single variant level using the methods for single SNP-based association studies. Total RNA was isolated from the mature xylem of 435 unrelated individuals of *P. tomentosa* in 2015 using the methods described above. RT-qPCR was conducted to assess the expression profiles of *Pto-ARF8* in mature xylem in the 435 unrelated individuals of *P. tomentosa*, which were used for gene expression-based association analysis.

### Transcript analysis of SNP genotypes

To investigate the effects of SNPs in the pre-miRNA region of *Pto-MIR167a*, RNAfold (http://rna.tbi.univie.ac.at/cgi-bin/RNAWebSuite/RNAfold.cgi) was used to predict secondary structures, and RT-qPCR was performed to investigate the transcript abundance of different genotypes of *Pto-MIR167a* in 10 individuals randomly selected from each genotype class in the association population. Additionally, the corresponding expression of targets lncRNA *ARFRL* and *Pto-ARF8* were measured in the background of different genotypes of the common SNPs in *Pto-MIR167a*. The differential expression of different genotypes was evaluated using analysis of variance (ANOVA).

## Results

### Identification of *Pto-MIR167a* and its potential targets, lncRNA *ARFRL*, and *Pto-ARF8*, in *P. tomentosa*

**Figure 1:**
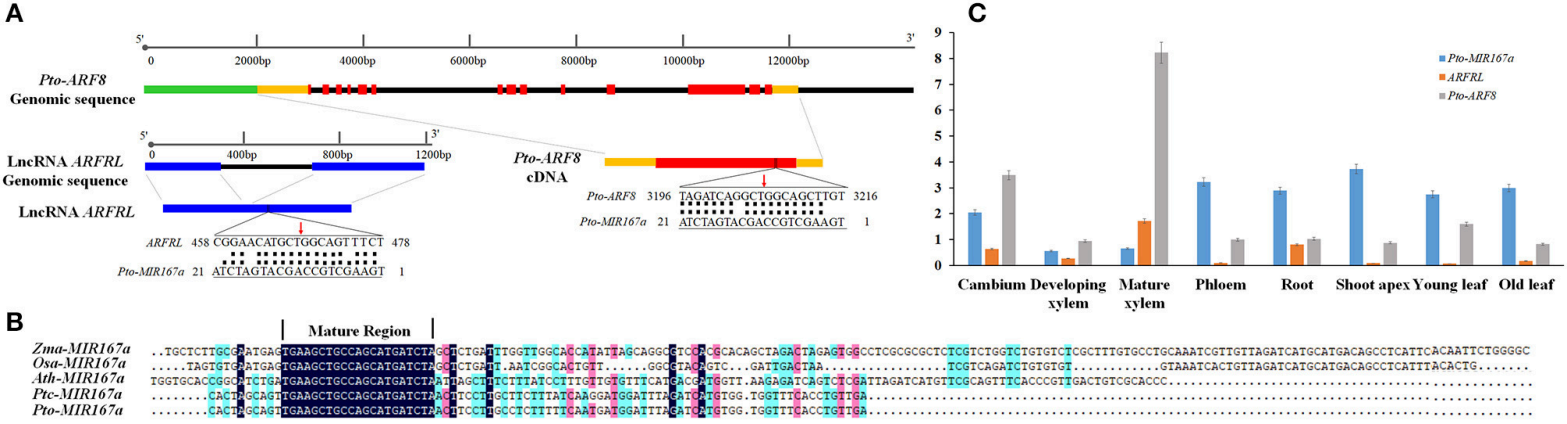
Characterization and expression analysis of *Pto-MIR167a* and its targets, lncRNA *ARFRL*, and *Pto-ARF8*. **(A)** The structures of the *Pto-ARF8* gene and long non-coding RNA (lncRNA) gene *ARFRL*, and the binding sites of Pto-miR167a with lncRNA *ARFRL* and *Pto-ARF8*. Green, orange, red, blue, and black lines represent the promoters, 5′/3′ untranslated regions (UTRs), exons, lncRNA transcripts, and introns/flanking regions, respectively. Red arrows represent the cleavage sites confirmed by 5′ rapid amplification of cDNA ends (5′-RACE). **(B)** Sequence alignment of the pre-miRNA region of the *MIR167a* genes of *Zea mays* (*Zma*), *Oryza sativa* (*Osa*), *Arabidopsis thaliana* (*Ath*), *Populus trichocarpa* (*Ptc*), and *P. tomentosa* (*Pto*). **(C)** The relative expression levels (arbitrary units normalized to the control) of *Pto-MIR167a* and its targets, lncRNA *ARFRL*, and *Pto-ARF8*, in eight tissues: cambium, developing xylem, mature xylem, phloem, root, shoot apex, young leaf, and old leaf, assessed by RT-qPCR with *Actin* as the internal control.

To identify lncRNAs with potential sponge roles for miR167 in *P. tomentosa*, we first cloned the pre-miRNA sequence of eight members of the *Pto-MIR167* gene family based on *Ptc-MIR167* pre-miRNA sequences in miRbase. Using the lncRNA database of cambium, developing xylem, and mature xylem of *P. tomentosa*, we identified lncRNA *ARFRL* (expectation = 2.5) as a potential target of Pto-miR167a-d, whose mature sequences were conserved in the Pto-miR167 family (Figure 1A). Sequencing yielded a 799-nt transcript sequence of lncRNA *ARFRL*. Sequence alignment of *Pto-MIR167a-d* in 43 unrelated individuals revealed that *Pto-MIR167a* had the nucleotide variations in its pre-miRNA region, thus we used *Pto-miR167a* for further analyses. Then, we cloned the 2089-bp genomic sequence of *Pto-MIR167a* primary transcript, containing an 89-bp pre-miRNA sequence with a 20-bp mature sequence and 1000-bp flanking sequence on each side of the pre-miRNA region. Secondary structure prediction for *Pto-MIR167a* pre-miRNA revealed a typical hairpin structure, confirming that Pto-miR167a is a miRNA. Alignment of the *Pto-MIR167a* pre-miRNA sequence with homologous miRNAs from *Arabidopsis thaliana, Oryza sativa, Zea mays*, and *P. trichocarpa* revealed that the mature sequences were completely conserved across the five species, wherase the pre-miRNA region displayed variable sequence identity (21.05–96.93%) and length (89–190 bp) (Figure 1B). We also cloned the 1190-bp genomic sequence of lncRNA gene *ARFRL*, including a 799-bp lncRNA transcribed sequence with an intron of 391-bp (Figure 1A).

Furthermore, psRNATarget and degradome sequencing were used to determine the targets of Pto-miR167a in 3000 cDNA sequences from the cDNA library of mature xylem of *P. tomentosa*, and *Pto-ARF8* was identified as the target gene of Pto-miR167a (Figure 1A and Figure S1). The cDNA clone of *Pto-ARF8* (MG873933) was 3854-bp in length with a 2490-bp open reading frame, 904-bp of 5′ untranslated region (UTR), and 406-bp of 3′ UTR. The encoded proteins displayed high protein similarity (98.92%) to *Ptc-ARF8* (Potri.004G078200) in *P. trichocarpa* and contained the conserved domain of the Auxin response factor (at amino acid residues 216–338), B3 DNA binding domain (at amino acid residues 129–231), and the AUX/IAA family domain (at amino acid residues 719–791). Interestingly, *Pto-ARF8* was also predicted as the potential *trans*-target of lncRNA *ARFRL* (*E*-value = −90.4).

To validate the regulatory roles of Pto-miR167a and its targets, 5′-RACE was conducted to confirm the cleavage sites of Pto-miR167a on lncRNA *ARFRL* and *Pto-ARF8*. The results revealed that both targets were supported by 5′-RACE. The cleavage site of Pto-miR167a in lncRNA *ARFRL* was at 468 nt, and the alignment range of Pto-miR167a and *Pto-ARF8* was at 3196–3216 nt with the cleavage site at 3206 nt, which was consistent with the degradome sequencing results (Figure 1A).

### Expression abundance of *Pto-MIR167a* and its targets, lncRNA *ARFRL*, and *Pto-ARF8*

To test the expression profiles of the three candidate genes, RT-qPCR was conducted to measure the transcript abundance in eight tissues and organs of *P. tomentosa*. The three genes exhibited distinct but partially overlapping expression patterns among the eight tissues and organs (Figure 1C). *Pto-MIR167a* was predominantly expressed the in shoot apex and phloem, and had low expression in mature xylem and developing xylem. In contrast, the target lncRNA *ARFRL* was preferentially expressed in mature xylem and had low expression in shoot apex and young leaf. *Pto-ARF8* expression peaked in mature xylem, and had low expression in shoot apex and old leaf, indicating that *Pto-ARF8* may be involved in wood formation. Correlation analysis indicated that *Pto-MIR167a* was significantly negatively correlated with lncRNA *ARFRL* and *Pto-ARF8* (*r* = −0.60 and −0.61, respectively, *P* < 0.01), suggesting that Pto-miR167a triggered the degradome of *ARFRL* and *Pto-ARF8*. Notably, lncRNA *ARFRL* and *Pto-ARF8* was significantly positively correlated (*r* = 0.90, *P* < 0.01) in the tested tissues, suggesting the potential sponge role of lncRNA *ARFRL* for Pto-miR167a in regulating the expression of *Pto-ARF8*. These results supported the regulatory roles of the miR167a-*ARFRL*-*ARF8* network during the process of growth and wood formation.

### Nucleotide diversity and LD analysis of the three candidate genes

**Figure 2:**
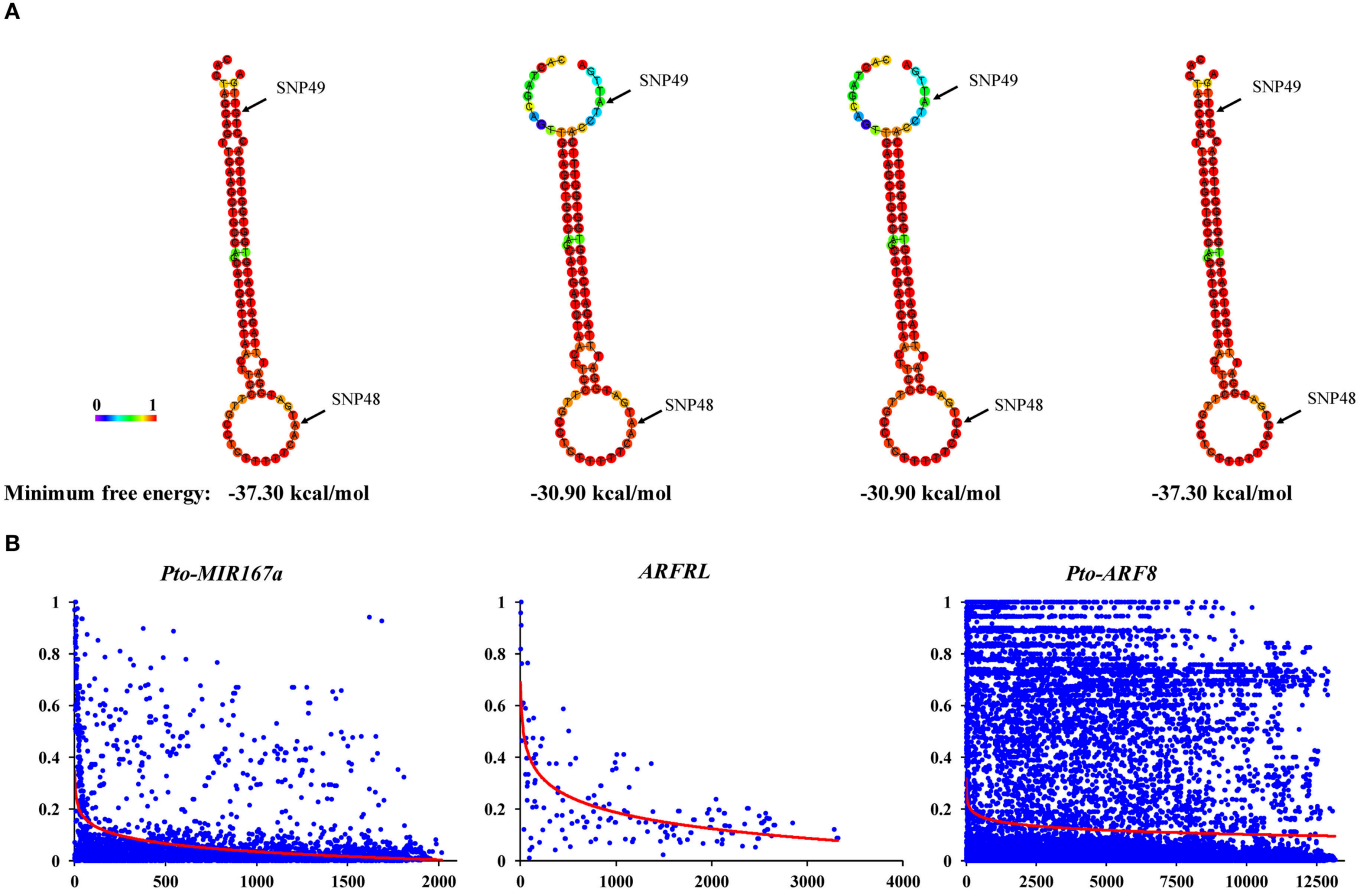
Secondary structures of the pre-miRNA region of *Pto-MIR167a* with single nucleotide polymorphisms (SNPs), and linkage disequilibrium within *Pto-MIR167a*, lncRNA gene *ARFRL*, and *Pto-ARF8*. **(A)** The secondary structures of *Pto-MIR167a* pre-miRNA sequence affected by two SNPs in the pre-miRNA region, and the minimum free energy of these secondary structures. **(B)** Pairwise correlations between SNPs (*r*^2^) are plotted against the physical distance between each SNP pair. The red curves indicate the nonlinear regressions of *r*^2^ on the physical distance in base pairs.

To identify SNPs within the candidate genes for association studies, we sequenced the 2089-bp genomic region of *Pto-MIR167a* primary transcript and the genes encoding lncRNA *ARFRL* and *Pto-ARF8*, including their 2000-bp promoters and 2000-bp flanking regions (downstream of the 3′ UTR), in 43 unrelated individuals from the association population (Table 1). For *Pto-MIR167a*, we detected 98 SNPs with an average frequency of 1/21 bp (π = 0.01806), of which 93 SNPs were common SNPs (MAF ≥ 5%). Two common SNPs were identified in the pre-miRNA region of *Pto-MIR167a*, with no SNPs in mature sequence, indicating that the mature region was the most highly conserved. Remarkably, we predicted the secondary structure of *Pto-MIR167a* based on the two common SNPs in the pre-miRNA region (SNP48 and SNP49). The results revealed that Pto-MIR167a_SNP49 in the pre-miRNA of *Pto-MIR167a* significantly altered the stem-loop structure and the minimum free energy (from −37.30 to −30.90 kcal/mol) of the predicted secondary structure of *Pto-MIR167a* (Figure 2A), indicating the crucial functions of Pto-MIR167a_SNP49 in *Pto-MIR167a*.

**Table 1:** Summary of single nucleotide polymorphisms (SNPs) of *Pto-MIR167a*, lncRNA gene *ARFRL*, and *Pto-ARF8*.

**Gene**	**Region**	**Length (bp)**	**Number of polymorphic sites**	**Number of common SNPs**	**Percentage polymorphisms (%)**	**Nucleotide diversity**	
						**π**	**θw**
***Pto-MIR167a***							
	Flanking region	2000	95	91	4.75	0.01832	0.01098
	Pre-mature region	89	3	2	3.37	0.01219	0.00779
	Mature region	21	0	0	0	0	0
	Total	2089	98	93	4.69	0.01806	0.01084
***Pto-ARFRL***							
	Promoter	2000	4	4	0.20	0.00122	0.00097
	LncRNA transcribed region	1190	5	5	0.42	0.00052	0.00046
	Flanking	2000	14	14	0.70	0.00189	0.00179
	Total	5190	23	23	0.44	0.00119	0.00106
***Pto-ARF8***							
	Promoter	2000	35	34	1.75	0.00385	0.00404
	5′ UTR	904	9	9	1.00	0.00435	0.00230
	Exon	2490	29	17	1.16	0.00246	0.00269
	Intron	5737	151	90	2.63	0.00656	0.00608
	3′ UTR	460	23	20	5.00	0.01650	0.01156
	Flanking	2000	100	95	5.00	0.01818	0.01156
	Non-synonymous	1911.3	10	7	0.52	0.00235	0.00290
	Synonymous	575.7	19	10	3.30	0.00284	0.00201
	Total silent^a^	11679.7	337	258	2.89	0.00812	0.00639
	Total^b^	13591	347	265	2.55	0.00731	0.00590

a Total silent: synonymous sites plus polymorphic sites in noncoding regions of genes.

b *Total: silent sites plus nonsynonymous sites of genes*.

In addition, 23 common SNPs were identified in the lncRNA gene of *ARFRL* gene with a low density of 1/260 bp (π = 0.00119), and five SNPs in the lncRNA transcribed sequences (π = 0.00052). For *Pto-ARF8*, 347 SNPs were detected across the entire gene with an average density of 1/39 bp (π = 0.00731), and the average non-synonymous (*d*_*N*_) nucleotide diversity was lower than that of synonymous (*d*_*S*_) nucleotide diversity, with the *d*_*N*_/*d*_*S*_ ratio (0.83) < 1 for exons within *Pto-ARF8*, indicating purifying selection for non-synonymous sites in the exons (Table 1). Among the 347 SNPs in *Pto-ARF8*, 265 SNPs were common SNPs (MAF ≥ 5%), and 93.58% of them were found in non-coding regions, including the promoter (34), 5′/3′ UTRs (29), introns (90), and flanking regions (95). Only 17 common SNPs were found in coding regions, with seven non-synonymous mutations (Table S5). Estimated *r*^2^ values for all SNP pairwise combinations, combined with their physical distance, were pooled to evaluate the overall patterns of LD for *Pto-MIR167a* and its targets, lncRNA *ARFRL*, and *Pto-ARF8*. Non-linear regression revealed a rapid decay, with *r*^2^ declining to 0.1 at distances of 251–13000 bp for each gene (Figure 2B), indicating that LD in the three candidate genes did not extend over the entire genes.

### Genetic dissection of allelic variation of *Pto-MIR167a* and its targets, lncRNA *ARFRL*, and *Pto-ARF8*, revealed by association studies

#### Single SNP-based association mapping

To further investigate the genetic effects of SNPs within *Pto-MIR167a, ARFRL*, and *Pto-ARF8* on tree growth and wood properties, we conducted 3810 association tests among the 381 common SNPs within the three candidate genes and 10 quantitative traits, using MLM in TASSEL 5.0. Collectively, 53 significant associations (*P* < 0.01, *Q* < 0.1) were detected, representing 41 unique SNPs in the three candidate genes and six traits (Table 2 and Table S6). The phenotypic variance (*R*^2^) explained by each association ranged from 9.11% (Pto-MIR167a_SNP80) to 23.76% (Pto-ARF8_SNP13), with an average of 14.73%. Four associations, Pto-MIR167a_SNP66 for DBH, Pto-ARF8_SNP13 for DBH, Pto-ARF8_SNP225 for HC, and Pto-ARF8_SNP227 for HC, explained more than 20% of the phenotypic variation (Table S6). Nine loci in *Pto-MIR167a* were significantly associated with DBH and V, including Pto-MIR167a_SNP49 in the pre-miRNA region associated with DBH with an *R*^2^ of 12.23%. Nine associations were tested among five unique SNPs in the lncRNA *ARFRF* gene and four traits (HEC, DBH, V, and FW), including one SNP (ARFRL_SNP7) in lncRNA transcribed sequences, indicating the pleiotropic effect of lncRNA *ARFRL* on tree growth and wood properties (Table 2 and Table S6). In addition, 27 SNPs from *Pto-ARF8* were associated with six traits, and Pto-ARF8_SNP96 in an exon caused the non-synonymous mutation of Gly to Asp, associated with MFA, with an *R*^2^ of 11.35%. These findings illustrated the common roles of the three genes in wood formation.

**Table 2:** Summary of significant SNPs within *Pto-MIR167a, ARFRL*, and *Pto-ARF8* associated with growth and wood properties in the association population of *P. tomentosa*.

**Traits**	**Number of association**	**Number of SNPs**			**Range of additive effects**	**Range of dominant effects**	**Range of *R*^2^ (%)**
		***Pto-ARF8***	***ARFRL***	***Pto-MIR167a***			
MFA	2	2	–	–	1.55	−3.99	10.45~11.35
V	9	4	2	3	7.63~24.63	−20.11~30.52	9.11~19.89
HC	20	20	–	–	5.30~9.24	−1.61~19.16	11.07~21.37
HEC	3	2	1	–	4.42~6.61	7.05~11.45	8.24~12.37
FW	3	1	2	–	0.80	3.25~11.69	10.28~15.11
DBH	16	4	3	9	2.53~14.43	−1.93~9.53	9.41~23.76
Total	53	33	8	12	0.80~24.63	−20.11~30.52	9.11~23.76

For the 53 SNP-trait associations, 75.47% exhibited additive effects and 88.68% showed dominant effects, and 64.15% (34) associations exhibited combined additive and dominant effects (Table 2 and Table S6). The additive effects for 40 significant associations ranged from 0.08 (ARFRL_SNP20) to 24.63 (Pto-ARF8-SNP159). Also, 47 significant associations with dominant effects ranging from −20.11 (Pto-ARF8_SNP159) to 30.52 (Pto-ARF8_SNP13) were identified, and 76.60% were positive dominant effects (Table S6). Interestingly, Pto-ARF8-SNP159 in the 3′ UTR had the minimum dominant effect and the largest additive effect for trait V. Remarkably, 12 unique SNPs from the three genes were associated with two traits with disparate additive and/or dominant effects and contributions to phenotypes, such as Pto-ARF8_SNP221 associated with HC and HEC with negative and positive dominant effects for HC (−2.61) and HEC (7.05), respectively, indicating the pleiotropy of the gene for the phenotypes. Additionally, each trait was associated with 2–20 SNPs from these three genes (Table 2 and Table S6). For example, 16 SNPs representing three SNPs in *Pto-MIR167a*, two SNPs in *ARFRL*, and four SNPs in *Pto-ARF8*, were simultaneously associated with V, with distinct genetic effects and contributions for the traits. These results indicated that these three genetic factors, *Pto-MIR167a*, lncRNA *ARFRL*, and *Pto-ARF8*, have the joint contributions to tree growth and wood properties through multiple aspects, and possess different effects for the specific traits.

#### Haplotype-based association analysis

**Figure 3:**
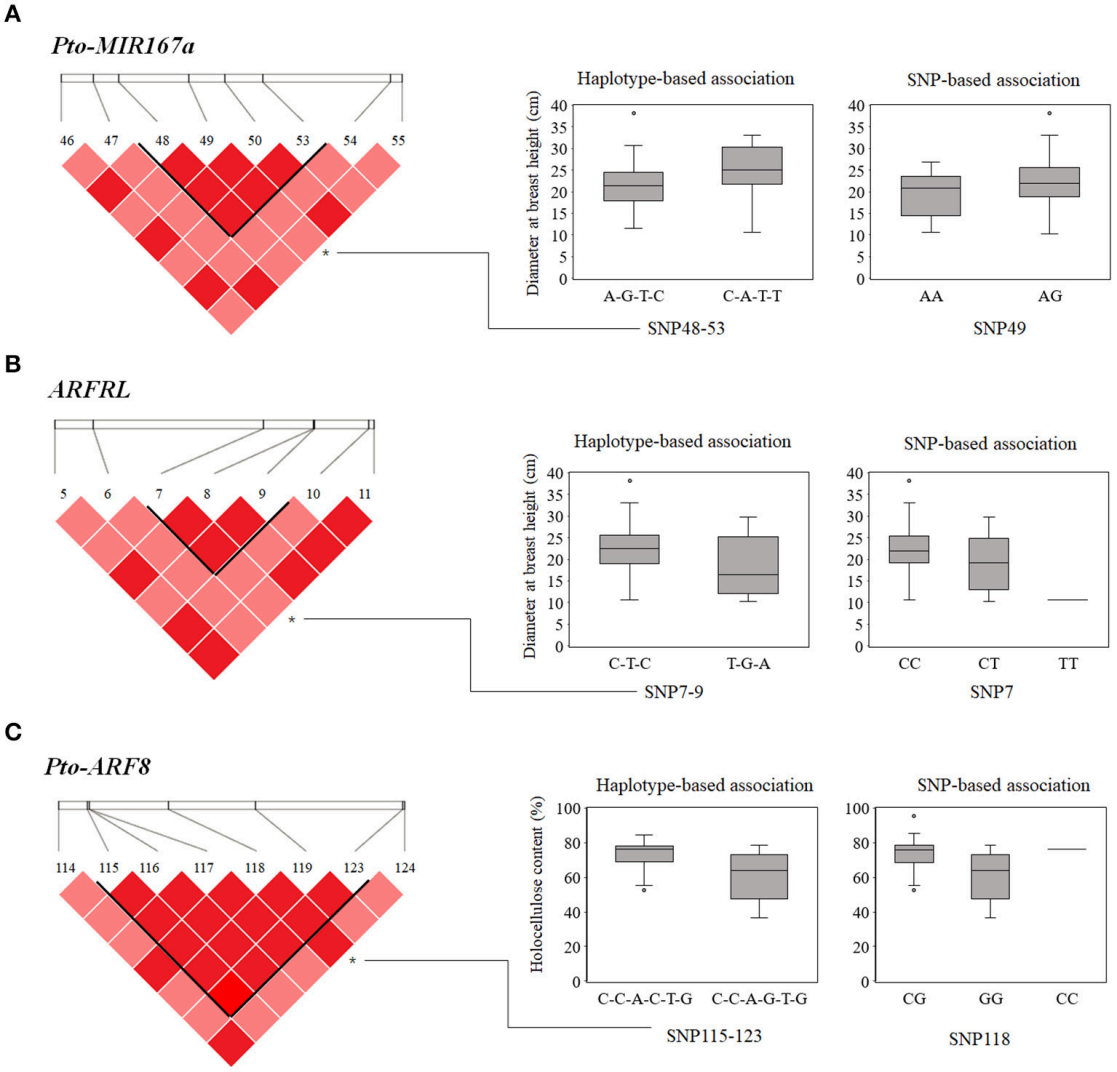
SNP-based and haplotype-based associations in the association population of *P. tomentosa*. The genotypic effects of the significant haplotypes of Pto-MIR167a_SNP48-53 **(A)**, lncRNA ARFRL_SNP7-9 **(B)**, and Pto-ARF8_SNP115-123 **(C)**, along with the single locus effects of Pto-MIR167a_SNP49, ARFRL_SNP7, and Pto-ARF8_SNP118, respectively, consistent with SNP-based associations.

Based on the LD of SNP pairs for each gene, we detected 70 common haplotypes (frequency ≥ 5%) from 32 high-LD blocks (*r*^2^ > 0.7, *P* < 0.01) within *Pto-MIR167a* and its targets lncRNA *ARFRL* and *Pto-ARF8*. Each gene contained 5–18 LD blocks and each block was composed of 2–4 common haplotypes (Table 3). Haplotype-based associations detected 44 significant haplotypes associated with 10 traits with *R*^2^ ranging from 0.39 to 9.12% (Table 3 and Table S7). Each trait was associated with 1–12 haplotypes from the three genes. For example, nine common haplotypes, including two in *Pto-MIR167a*, two in lncRNA gene *ARFRL*, and five in *Pto-ARF8*, were simultaneously associated with HC, with *R*^2^ ranging from 1.65 to 3.89%. In addition, 15 associated haplotypes were shared among the traits. For example, both the haplotype C-A-T-T from SNP48-53 in *Pto-MIR167a* and the haplotype C-T-C in SNP7-9 of *ARFRL* were associated with DBH and V, and the haplotype C-C-A-G-T-G in SNP115-123 of *Pto-ARF8* was associated with HC and DBH (Figure 3). Notably, the three haplotype-based associations were also strongly supported by single SNP-based associations (Pto-MIR167a_SNP49, ARFRL_SNP7, and Pto-ARF8_SNP118) for the same traits (Figure 3).

**Table 3:** Summary of significant haplotypes within *Pto-MIR167a*, lncRNA *ARFRL*, and *Pto-ARF8* associated with growth and wood properties in the association population of *P. tomentosa*.

**Gene**	**Number of LD blocks**	**Number of common haplotypes^a^**	**Length range of haplotypes^b^**	**Number of associated haplotypes^c^**	**Associated traits**	**Range of *R*^2^ (%)**
*Pto-MIR167a*	9	20	2–6	11	CC, DBH, FW, HC, V	0.95–9.12
*ARFRL*	5	10	2–3	8	DBH, FW, HC, HEC, V	0.66–9.10
*Pto-ARF8*	18	40	2–7	25	CC, DBH, FL, FW, H, HC, HEC, LC, MFA, V	0.39–7.67
Total	32	70	2–7	44	CC, DBH, FL, FW, H, HC, HEC, LC, MFA, V	0.39–9.12

a common haplotype: frequency ≥ 0.05.

b *length range of haplotypes: one SNP as a unit*.

c *associated haplotypes: the significant level for association with P < 0.01*.

### Genetic interactions of *Pto-MIR167a* and its targets, lncRNA *ARFRL*, and *Pto-ARF8*, revealed by epistasis model

**Figure 4:**
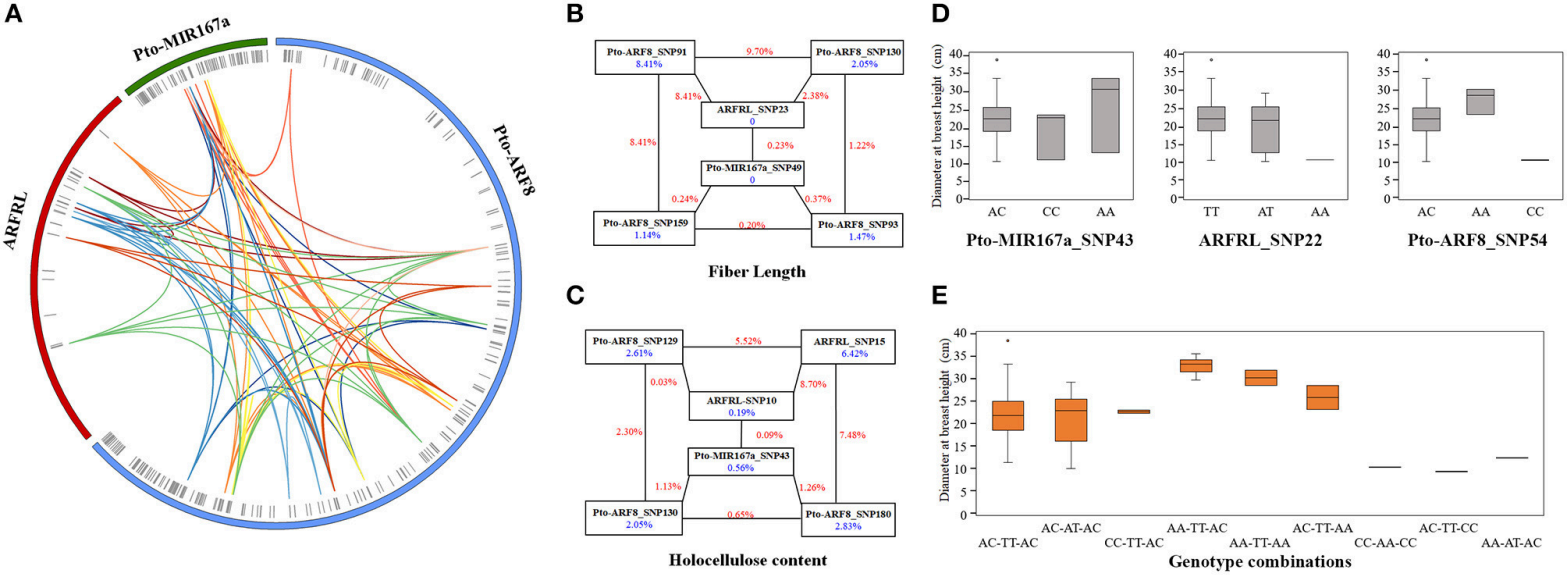
Epistatic network among the SNPs in *Pto-MIR167a* and its targets, lncRNA gene *ARFRL*, and *Pto-ARF8*, and their epistatic effects on traits. **(A)** Structural network revealing the epistatic interactions of different loci of *Pto-MIR167a*, lncRNA gene *ARFRL*, and *Pto-ARF8*. The outer circles represent the three different genes. The middle circles indicate the SNP positions within the genes. The inner lines represent the pairwise interactions, with different colored lines representing for different traits, i.e., deep red, light blue, pink, orange, yellow, green, deep blue, red, blue, and deep orange indicate diameter at breast height, tree height, stem volume, fiber length, fiber width, microfibril angle, lignin content, α-cellulose content, holocellulose content, and hemicellulose content, respectively. **(B,C)** Interaction graphs for fiber length and holocellulose content among the SNPs of the three candidate genes. The blue values in boxes represent the single-marker effects, and the red values along the lines indicate pairwise effects. **(D)** Box plots depicting the single-locus effects of different genotypes of the three SNPs for phenotypic variations. **(E)** Boxes displaying the phenotypic values of different genotypic combinations of the three SNPs, Pto-MIR167a_SNP43, ARFRL_SNP22, and Pto-ARF8_SNP54.

To decipher the genetic networks of *Pto-MIR167a* and its targets lncRNA *ARFRL* and *Pto-ARF8*, MDR 3.0.2 was conducted to investigate the pairwise effects of three genes for tree growth and wood properties. We detected a total of 88 pairwise interactions (*P* < 0.01, *Q* < 0.1), representing 33 unique SNPs from *Pto-MIR167a* (6), lncRNA gene *ARFRL* (9), and *Pto-ARF8* (18), with 10 traits (Figure 4A and Table S8). Single effects for each associated SNP ranged from 0 to 8.41% and pairwise effects from 0.03 to 9.70%. The pairwise epistatic effects, assessed by IG, ranged from −6.64 to 3.45%, and 84.10% of the SNP-SNP associations were negative IGs (Table 4 and Table S8), representing the redundant functions of the two loci for associated traits. For the 88 pairwise interactions, 53 SNP-SNP associations represented the epistatic interactions between genes, including 50.94% for lncRNA-mRNA, 39.62% for miRNA-mRNA, and 9.43% for miRNA-lncRNA (Figure 4A and Table S8). Only 12.12% of the associated loci were detectable with additive and dominant effects, indicating that epistasis captured SNPs with minor effects. Such as Pto-ARF8_SNP93 had joint additive, dominant, and epistatic genetic effects for the phenotypes (Tables S6, S8). In addition, 11 SNPs detected epistatic interactions with multiple SNPs for more than one trait with different effects (Figure 4A). For example, Pto-ARF8_SNP54 formed six pairwise effects with six SNPs from *Pto-MIR167a* (2) and *ARFRL* (4) for the DBH, MFA, and V traits, with pairwise effects of 0.09–7.39% (Table S8).

**Table 4:** Summary of significant SNP pairs associated with 10 tree growth and wood properties in *P. tomentosa*.

**Traits**	**Number of interactions**	**Number of SNPs**			**Interaction effects (%)**	**IGs(%)**
		***Pto-MIR167a***	***ARFRL***	***Pto-ARF8***		
CC	10	3	–	2	0.28~2.38	−4.28~0.59
DBH	6	1	2	1	0.07~4.68	−6.64~3.45
FL	15	1	1	4	0.08~9.70	−2.42~0.32
FW	10	1	–	4	0.31~9.70	−3.49~−0.21
H	6	–	2	2	0.03~5.51	−0.60~2.12
HC	15	1	2	3	0.03~8.70	−4.94~2.10
HEC	6	–	1	3	0.15~5.90	−5.74~−2.20
LC	6	1	–	3	0.11~3.48	−3.51~0.04
MFA	15	–	2	4	0.09~7.39	−4.57~2.32
V	3	1	–	2	2.30~3.48	−3.57~−1.22

To investigate the pairwise effects for tree growth and wood properties, interaction graphs for FL and HC were constructed (Figures 4B,C). These revealed two-way interactions between six SNPs from the three candidate genes, indicating the genetic networks of the three genes for wood formation traits. The pairwise effects between the six SNPs for FL ranged from 0.08 to 9.70%. Interestingly, ARFRL_SNP23 and Pto-MIR167a_SNP49 had no effects on FL, while they did have epistatic effects on FL when combined with other SNPs, with pairwise effects of 0.08–8.41% (Figure 4B and Table S8). Additionally, with regard to 16 interactions for FL and HC, only three pairwise interactions had positive IGs for traits, Pto-MIR167a_SNP49-ARFRL_SNP23 and ARFRL_SNP23-Pto-ARF8_SNP130 for FL, and ARFRL_SNP10-ARFRL_SNP15 for HC (Figures 4B,C and Table S8), indicating that the pairwise effects of the two loci were higher than the sum of the effects of the single locus for the traits. Remarkably, loci with epistatic effects on traits were dependent on different genotype combinations. As shown in Figures 4D,E, Pto-MIR167a_SNP43, ARFRL_SNP22, and Pto-ARF8_SNP54 were detected with epistatic interactions for DBH, and the different genotype combinations of the three loci differed significantly from the values of the single locus. The genotype combinations of AA-TT-AC and AC-TT-CC represented the maximum and minimum values for DBH (Figure 4E). These findings reflected the network interactions of the three genes for tree growth and wood properties, and demonstrated that they affected phenotypic variations by different genotype combinations of significant loci.

### Genetic regulation of tree growth and wood properties through regulation of the expression of *Pto-ARF8* revealed by gene expression-based association analysis

**Figure 5:**
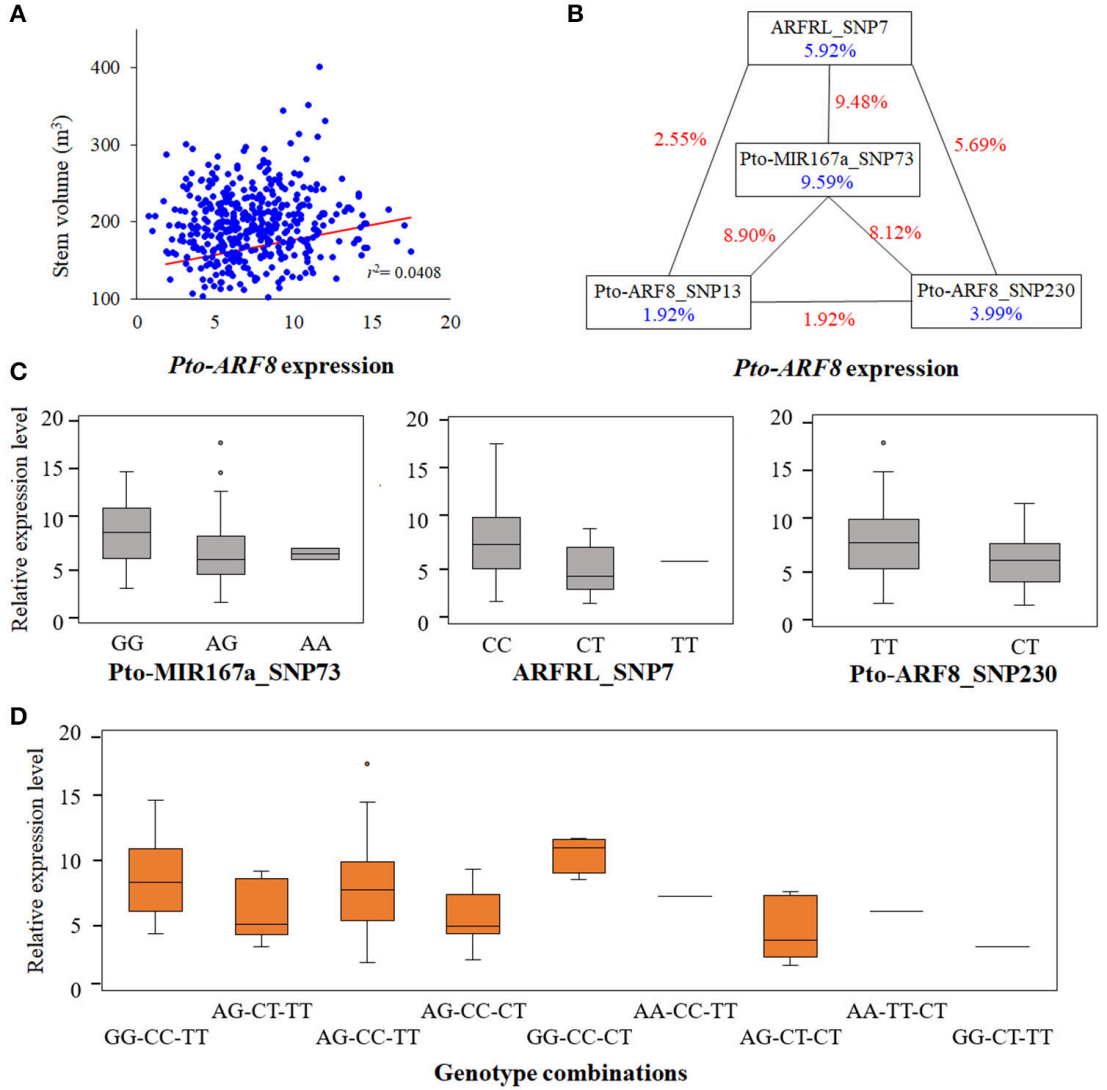
Epistatic interactions of significant SNPs in *Pto-ARF8* expression variation. **(A)** Correlation between stem volume variation and *Pto-ARF8* expression levels detected by RT-qPCR. **(B)** Interaction graph of *Pto-ARF8* expression among four significant SNPs in the three candidate genes. The blue values in boxes represent single-marker effects and the red values along the lines indicate the pairwise epistatic effects. **(C)** Box plots revealing the single-locus effects of different genotypes for expression traits of *Pto-ARF8*. **(D)** Boxes displaying the expression variation of different genotype combinations of three SNPs, Pto-MIR167a_SNP73, ARFRL_SNP7, and Pto-ARF8_SNP230.

According to the results of the single SNP-based association studies, we identified significant associations between SNPs within *Pto-ARF8* and six traits (V, DBH, FW, MFA, HC, and HEC), and the expression of *Pto-ARF8* was positively correlated with trait V (*r* = 0.202, *P* < 0.01), indicating that *Pto-ARF8* may affect phenotypic variations of V via its expression to some extent, as an alternative pathway (Figure 5A). Thus, we investigated the potential genetic effects of SNPs within the three candidate genes for *Pto-ARF8*. In total, five significant SNPs were detected to have genetic effects on the expression of *Pto-ARF8* (*P* < 0.01, *Q* < 0.1), with *R*^2^ of 8.58–15.68%, including two significant loci in SNP-based association mapping, ARFRL_SNP7 and Pto-ARF8_SNP13 (Table S9). Interestingly, four SNPs (Pto-MIR167a_SNP73, ARFRL_SNP7, Pto-ARF8_SNP13, and Pto-ARF8_SNP230) formed an epistatic interaction network for the expression of *Pto-ARF8* (Figure 5B). The genotype combinations of GG-CC-CT and GG-CT-TT for Pto-MIR167a_SNP73, ARFRL_SNP7, and Pto-ARF8_SNP230 had the largest and smallest contributions to the expression of *Pto-ARF8*, respectively (Figures 5C,D). The results illustrated an alternative regulatory model by which the significant loci within the three genes may affect phenotypes by regulating the expression of *Pto-ARF8*.

### Transcript analysis of significant SNP genotypes of *Pto-MIR167a*

To further explore the effects of significant SNPs of Pto-MIR167a_SNP49 in the pre-miRNA region on the transcript abundance of *Pto-MIR167a*, RT-qPCR was performed to test the relative expression levels of *Pto-MIR167a* in mature xylem from *P. tomentosa*, which was randomly chosen from 10 individuals for each genotype class of Pto-MIR167a_SNP49. The transcript abundance of *Pto-MIR167a* significantly changed across the different genotypes, with high expression levels in the AG group (1.228 ± 0.041, arbitrary units normalized to the control), and low expression in the AA group (0.970 ± 0.036) (Figure S2). Next, we also tested the corresponding expression of lncRNA *ARFRL* and *Pto-ARF8*, and found that *Pto-MIR167a* suppressed the expression of lncRNA *ARFRL* and *Pto-ARF8*. The expression levels of lncRNA *ARFRL* and *Pto-ARF8* were higher in the AA group (1.255 ± 0.039 and 6.189 ± 0.042, respectively) than in the CC group (0.953 ± 0.023 and 5.104 ± 0.035, respectively) (Figure S2).

## Discussion

### Characterization of *Pto-MIR167a* and its network with targets of lncRNA *ARFRL* and *Pto-ARF8* in *P. tomentosa*

Tree growth and wood formation traits are regulated by coordinated networks involving multiple genetic factors, e.g., transcription factors, lncRNAs, and miRNAs (Kirst et al., 2004; Neale and Kremer, 2011). Among these factors, miRNAs and lncRNAs are essential regulators that cooperate with their corresponding targets, leading to phenotypic variations (Ramachandran and Chen, 2008; Ponting et al., 2009). SNPs in pre-miRNA have been reported to cause functional changes in miRNA, mainly via influencing pre-miRNA secondary structures and affecting the abundance of mature miRNA, thus leading to phenotypic variation (Ryan et al., 2010). For instance, SNPs identified in the pre-miRNA of the *Pto-MIR160a* changed the stem-loop number of *Pto-MIR160a* secondary structure and affected its stability, ultimately altering the transcript abundance of miRNA genes (Tian et al., 2016a). Our findings revealed that the common SNP Pto-MIR167a_SNP49 in the pre-miRNA of *Pto-MIR167a* altered the stem-loop structure and affected the stabilization of the secondary structure, and the minimum free energy changed from −37.30 to −30.90 kcal/mol (Figure 2A). Additionally, the transcript abundance of *Pto-MIR167a* also varied across the different genotypes of Pto-MIR167a_SNP49 (Figure S2), illustrating that SNPs in the pre-miRNA region affect the transcription of *Pto-MIR167a*. Beyond that, the expression levels of lncRNA *ARFRL* and *Pto-ARF8* were also altered by the different genotypes of Pto-MIR167a_SNP49, supporting the hypothesis that the alteration stability of the secondary structure affects the accumulation of mature miRNA, thus impairing the regulation of miRNA targets (Duan et al., 2007). Moreover, Pto-MIR167a_SNP49 was also identified as associating with DBH under SNP-based and haplotype-based associations (Figure 3 and Tables S6, S7). Taken together, these results illustrate the vital roles of SNPs in the pre-miRNA region on miRNA biogenesis and their effects on phenotypes, possibly by regulating the expression of target genes (Sun et al., 2009).

We also identified 91 common SNPs in the flanking region of *Pto-MIR167a*, where SNPs may affect pre-miRNA formation (Zeng and Cullen, 2005). Remarkably, no SNPs were identified in the mature region (Table 1), and the *Pto-MIR167a* pre-miRNA sequence alignment with *A. thaliana, O. sativa, Z. mays*, and *P. trichocarpa* revealed complete conservation of the mature region (Figure 1B), strongly supporting the idea that the miRNA mature sequence is highly conserved so that it can maintain its functions across multiple species (Voinnet, 2009). Tissue-specific analysis revealed variable abundance of *Pto-MIR167a* in stem tissue (phloem, developing xylem, mature xylem, and cambium) (Figure 1C), indicating its regulatory roles in wood formation. In contrast, the high abundance of lncRNA *ARFRL* and *Pto-ARF8* in mature xylem revealed that they may directly participate in the secondary cell wall formation process (Figure 1C). Expression correlation analysis revealed negative expression correlations between *Pto-MIR167a* and lncRNA *ARFRL* (*r* = −0.60, *P* < 0.01) and *Pto-ARF8* (*r* = −0.61, *P* < 0.01) in eight tested tissues, illustrating that the regulatory network of the three genes may affect tree growth and wood properties through a shared pathway.

For lncRNA *ARFRL*, only 23 SNPs were identified with a nucleotide diversity of π = 0.00119, which was lower than that of lncRNA *UGTRL* (π = 0.02607) previously reported in *P. tomentosa* (Quan et al., 2016). It is probable that the origin of lncRNAs is complex, and some lncRNAs partially overlap or derive from the exons of protein-coding genes (Ponting et al., 2009), where the sequences are more conserved than in non-coding regions. We also identified the Pto-miR167a binding sites in lncRNA *ARFRL* (Figure 1A), and predicted lncRNA *ARFRL* as the target of Pto-miR167a with an expectation of 2.5. Expression correlation also exhibited a strong positive correlation between lncRNA *ARFRL* and *Pto-ARF8* (*r* = 0.90, *P* < 0.01). These results strongly support the sponge role of lncRNA *ARFRL* for Pto-miR167a in regulating the expression of *Pto-ARF8*. However, the detailed mechanisms of sponge lncRNA *ARFRL* will require further investigation in the future. Importantly, the expression and sequence characteristics of the three genes offer strong functional evidence of the miR167a-*ARFRL*-*ARF8* network in tree growth and wood formation.

### SNPs within *Pto-MIR167a* and its targets lncRNA *ARFRL* and *Pto-ARF8* are associated with tree growth and wood properties

In this study, we identified 53 and 43 significant associations (*P* < 0.01) according to SNP-based association studies and haplotype-based association studies, respectively, suggesting that the three candidate genes share common functions in tree growth and wood properties (Tables 2, 3). In total, nine SNPs in *Pto-MIR167a* were significantly associated with traits DBH and V, and three loci (SNP49, SNP66, and SNP69) were also identified in haplotype-based association studies for the same traits, which supported the SNP-based associations (Figure 3A). Moreover, eight SNPs in the flanking region of *Pto-MIR167a* were associated with DBH and V, and eight haplotypes from SNPs in the flanking region were associated with five traits (CC, DBH, FW, HC, and V), indicating the functional roles of flanking regions for miRNA genes and suggesting that *Pto-MIR167a* may affect wood formation through multiple pathways. Correspondingly, nine associations were identified in lncRNA gene *ARFRL*, and only one significant SNP was found in the lncRNA transcribed sequences (ARFRL-SNP7), indicating that flanking regions in ncRNAs play a significant role in the regulation of gene expression and phenotypes (Zeng and Cullen, 2005).

In addition, the significant SNPs in *Pto-ARF8* affected phenotypic variations through different models. Pto-ARF8_SNP96 in an exon of *Pto-ARF8* caused the non-synonymous mutation of Gly to Asp, which was associated with MFA, indicating it may affect wood formation by changing the encoded amino acid. Also, SNPs in promoters and introns affect the phenotypes by affecting the transcription via various methods, such as altering transcription binding sites and processing signals (Greenwood and Kelsoe, 2003; Kimchi-Sarfaty et al., 2007).

A total of 53 associations were detected with additive and/or dominant effects on traits and offered detailed clues of the loci for traits, which provide the abundant resources for the genetic improvement of trees (Table S6). Interestingly, loci in the three genes exhibited different models for different traits. In total, 12 SNPs and 15 haplotypes from the three genes were associated with multiple traits with different additive and dominant effects and *R*^2^ values, such as Pto-ARF8_SNP221 for HC and HEC (Tables S6, S7), indicating the pleiotropy of the genetic factors for tree growth and wood formation. Each trait was associated with multiple SNPs or haplotypes from the three candidate genes. For example, three SNPs in *Pto-MIR167a*, two SNPs in *ARFRL*, and four SNPs in *Pto-ARF8* were simultaneously associated with V, harboring distinct genetic effects and *R*^2^, indicating the joint effects of the three genetic factors on phenotypes through a shared pathway. The different action models and effects of the associated loci for different traits demonstrated the pleiotropy of the three genetic factors, and enriched the functional understanding of *Pto-MIR167a* and its targets, lncRNA *ARFRL*, and *Pto-ARF8*, in wood formation of trees.

### The interaction of *Pto-MIR167a* and its targets lncRNA *ARFRL* and *Pto-ARF8* on phenotypes at genomic and transcriptional levels

The significant regulatory roles of the miR167-ARF system on growth and development in *Arabidopsis*, such as root architecture (Gifford et al., 2008) and reproduction (Wu et al., 2006), have been well characterized. In *Populus*, miR167 is also involved in various biotic and abiotic stress responses (Lu et al., 2008; Jia et al., 2009). However, the regulatory genetic interactions of miR167a with other genetic factors are largely unknown. Association studies (additive and dominant effects) and expression pattern analyses have revealed the common roles of *Pto-MIR167a* and its targets, lncRNA *ARFRL*, and *Pto-ARF8*, in tree growth and wood properties. Epistasis provided additional evidence of genetic interactions among multiple genes for quantitative traits, which offers effective and complementary information for breeding purposes (Mackay, 2014). Here, we identified 88 pairwise epistatic interactions, and 60.23% of the SNP-SNP pairs represented interactions between genes (Figure 4A), illustrating the genetic interactions among the three candidate genes for phenotypes. The majority of the epistatic interactions (84.10%) had negative IGs, indicating that the interactions among *Pto-MIR167a*, lncRNA gene *ARFRL*, and *Pto-ARF8* displayed functional redundancy in tree growth and wood properties. Notably, both the negative and positive IGs represented the close genetic interactions of miRNA-lncRNA-mRNA for traits (Moore et al., 2006).

Moreover, the interaction networks revealed that 11 SNPs detected epistatic interactions with multiple SNPs from different genes (Table S8), indicating the pleiotropy of functional SNPs for traits. The SNPs identified with the epistasis model were associated with different effects for traits, e.g., Pto-ARF8_SNP54, suggesting the complexity of the interactions between *Pto-MIR167a* and its targets, lncRNA *ARFRL*, and *Pto-ARF8*, for traits. Interestingly, the complicated network also reflected specific loci effects for traits, such as those of ARFRL_SNP23 and Pto-MIR167a_SNP49, which only the two loci possessed functional roles only when they formed pairwise epistatic interactions with other SNPs (Figure 4B). Remarkably, multiple SNPs with epistatic interactions affected phenotypes by different genotype combinations (Figures 4D,E). For example, the AA-TT-AC genotype combinations from Pto-MIR167a_SNP43, ARFRL_SNP22, and Pto-ARF8_SNP54 contributed the most to DBH, and also had stronger effects than single genotypes (Figure 4B), illustrating that genotype combinations with epistatic effects have more powerful effects than single loci.

In addition to their interactions at the genomic level, the genetic variants also affected phenotypes at the transcriptional level (Westra and Franke, 2014). In principle, the functional roles of ncRNA on phenotypes mainly depend on their targets (He and Hannon, 2004; Ponting et al., 2009). In our studies, the positive correlations of *Pto-ARF8* expression and trait V indicated that alternative mechanisms of significant genes affect phenotypes by regulating their own expression (Li et al., 2013) (Figure 5A). We identified five SNPs from three candidate genes significantly associated with *Pto-ARF8* expression (*R*^2^: 8.58–15.68%), including two significant loci identified by association mapping, ARFRL_SNP7 and Pto-ARF8_SNP13 (Table S9). These results illustrated that significant loci can affect phenotypes via regulating gene expression, thus affecting phenotype variations (Li et al., 2013). Among the SNPs associated with *Pto-ARF8* expression, one was from the miRNA gene and one was from the lncRNA gene, supporting the regulatory roles of *Pto-MIR167a* and lncRNA *ARFRL* on *Pto-ARF8* and illustrating that genetic variants in ncRNA genes are also indispensable for gene expression. Interestingly, three SNPs (Pto-MIR167a_SNP73, ARFRL_SNP7, and Pto-ARF8_SNP230) formed epistatic interaction networks for *Pto-ARF8* expression, and the genotype combinations contributed differentially to the expression of *Pto-ARF8*, which provides a valuable resource for applications. Based on the findings of the association analysis for physiology and expression traits, the roles of lncRNA ARFRL should not be neglected, and ARFRL_SNP7 exhibited its functionality in the regulation of wood formation, which should be validated in the future. In general, epistasis illustrated the interaction networks of miR167a-*ARFRL*-*ARF8* in trees, and gene expression-based association analysis aid in interpreting the genetic regulatory roles of the three genetic factors at the transcriptional level, providing an effective method for interpreting the genetic interactions of multiple genes for complex traits in trees.

## Conclusion

In our studies, we used expression profiles, association genetics (additive, dominant, and epistastic), and gene expression-based association analysis to investigate the allelic interactions within *Pto-MIR167a* and its target genes, sponge lncRNA *ARFRL*, and *Pto-ARF8*, in tree growth and wood formation. Expression pattern analysis revealed the potential function of regulatory networks of these three genes in wood formation. SNP-based and haplotype-based association studies provided genetic evidence for their common roles in tree growth and wood properties. Epistatic analysis uncovered the genetic interactions of the three genes and clarified the roles of the epistatic network in phenotypic variations. Notably, we also deciphered the significant variants within the three genetic factors contributing to phenotypes by regulating the expression of *Pto-ARF8*, revealed by gene expression-based association studies. Taken together, we investigated the potential roles of networks of *Pto-MIR167a*, lncRNA *ARFRL*, and *Pto-ARF8* in tree growth and wood formation, and proposed a feasible method for exploring the genetic interactions of miRNA-lncRNA-mRNA networks in the population genetics of trees.

## Data archiving statement

Sequence data in this article have been deposited with the GenBank Data Library under the accession numbers MG873890–MG874018, and the transcriptome sequencing for lncRNAs and degradome sequencing data are available in SRA database under the accession number SRP073689 and SRX1447192, respectively.

## Author contributions

DZ designed the conception and experiment. MQ and LX performed the experiments. FS, WL, and JS helped to collect and analyze the data. MQ wrote the manuscript. QD, XL, and DZ provided valuable suggestions on the manuscript. DZ obtained funding and is responsible for this article. All authors read and approved the manuscript.

### Conflict of interest statement

The authors declare that the research was conducted in the absence of any commercial or financial relationships that could be construed as a potential conflict of interest.

## Supplementary material

Figure S1 The most likely cleavage sites between Pto-miR167a and *Pto-ARF8*, identified by degradome sequencing.

Figure S2 Expression levels of *Pto-MIR167a* and its targets, lncRNA *ARFRL* and *Pto-ARF8*, on the background of different genotypes of Pto-MIR167a_SNP49.

Table S1 Phenotypic correlations of tree growth and wood property traits in the association population of *P. tomentosa* used in our studies.

Table S2 The miRNA-mRNA pairs identified by degradome sequencing.

Table S3 Gene-specific primers used in 5′ rapid amplification of cDNA ends (5′-RACE).

Table S4 Real-time PCR primers used in our studies.

Table S5 Common single nucleotide polymorphisms (SNPs; minor allele frequency ≥ 5%) in *Pto-MIR167a*, lncRNA gene *ARFRL*, and *Pto-ARF8* identified in our studies.

Table S6 Details of significant SNPs within candidate genes associated with growth and wood properties in the association population of *P. tomentosa*.

Table S7 Significant haplotypes from three candidate genes associated with growth and wood properties in the association population of *P. tomentosa*.

Table S8 SNP pairs and their main effects detected among the three candidate genes in the association population of *P. tomentosa*.

Table S9 Details of significant SNPs within candidate genes associated with expression levels of *Pto-ARF8* in the association population of *P. tomentosa*.
