# Supplementary figures and images for: Association Genetics in Populus Reveal the Allelic Interactions of Pto-MIR167a and Its Targets in Wood Formation
Source: Front Plant Sci. 2018 Jun 12;9:744. doi: 10.3389/fpls.2018.00744 (PMC6005902; doi:10.3389/fpls.2018.00744)

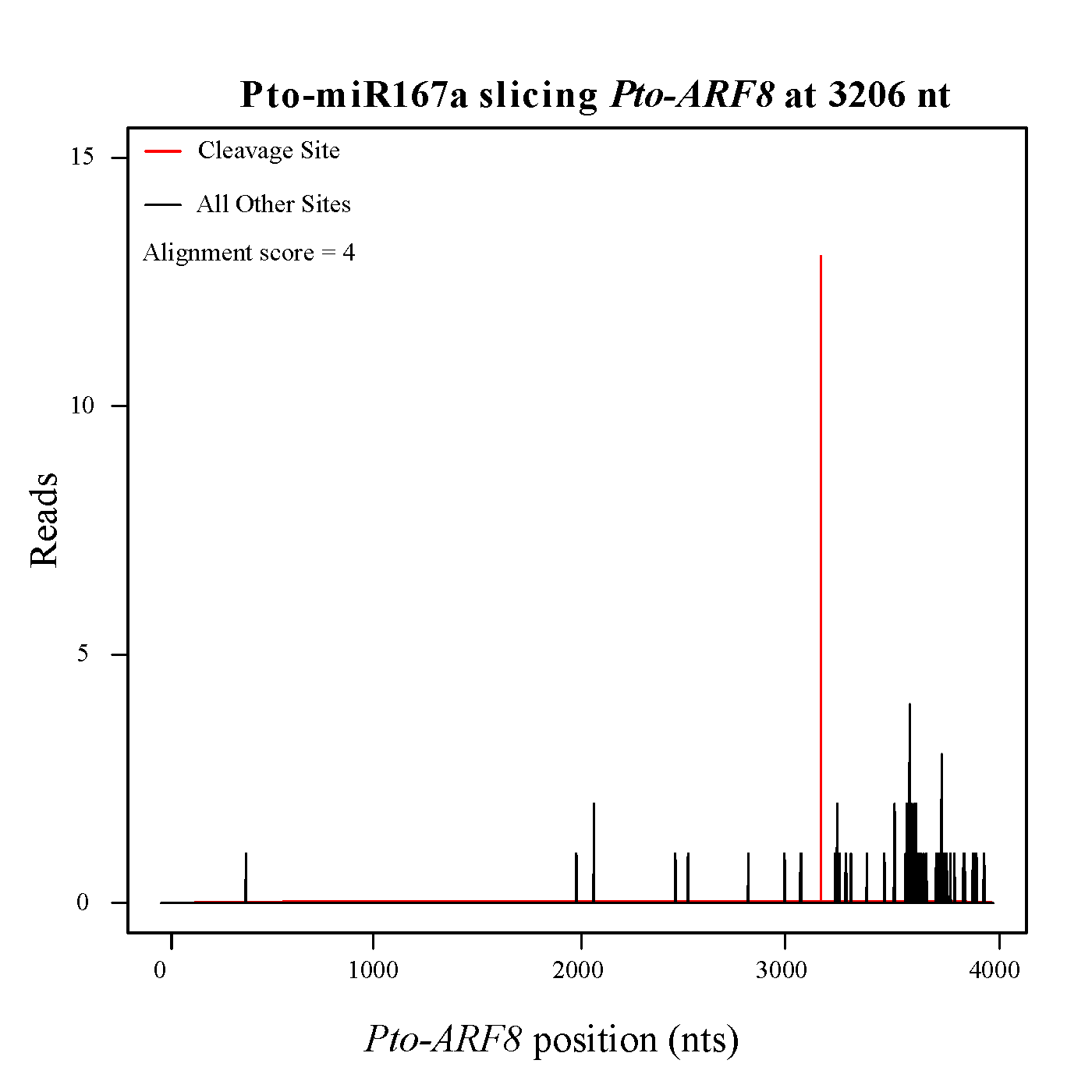

Supplement: Figure S1 — The most likely cleavage sites between Pto-miR167a and Pto-ARF8, identified by degradome sequencing. [file Image_1.TIFF]

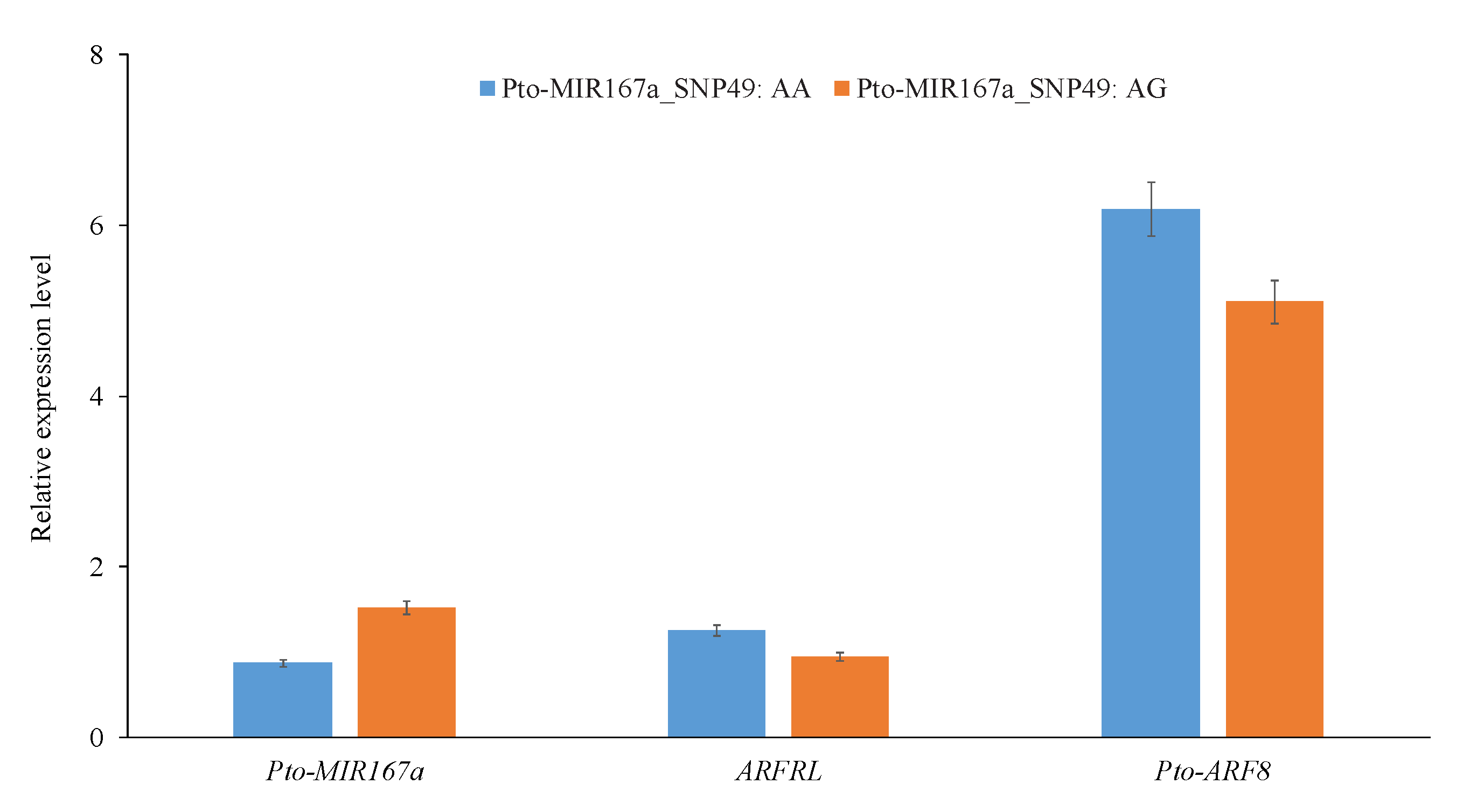

Supplement: Figure S2 — Expression levels of Pto-MIR167a and its targets, lncRNA ARFRL and Pto-ARF8, on the background of different genotypes of Pto-MIR167a_SNP49. [file Image_2.TIFF]
